# Molecular phylogeny and intraspecific differentiation of the *Trapelus agilis* species complex in Iran (Squamata: Agamidae) inferred from mitochondrial DNA sequences

**DOI:** 10.7717/peerj.8295

**Published:** 2020-02-17

**Authors:** Ali-Asghar Shahamat, Eskandar Rastegarpouyani, Nasrullah Rastegar-Pouyani, Seyyed Saeed Hosseinian Yousefkhani, Michael Wink

**Affiliations:** 1 Department of Biology, Faculty of Science, Razi University, Kermanshah, Iran; 2 Department of Biology, Faculty of Science, Hakim Sabzevari University, Sabzevar, Iran; 3 Iranian Plateau Herpetology Research Group (IPHRG), Faculty of Science, Razi University, Kermanshah, Iran; 4 Institute of Pharmacy and Molecular Biotechnology (IPMB), Heidelberg University, Heidelberg, Germany

**Keywords:** Miocene, *Trapelus sanguinolentus*, Zagros Mountain., Iranian Plateau, Agamidae, Western Asia

## Abstract

**Background:**

* Trapelus agilis* consists of different morphotypes with restricted distributions in the Iranian Plateau. The phylogeny of the species complex has not been resolved so far, but recently *Trapelus sanguinolentus* were elevated from this complex into a full species. Other populations of the species complex need to be evaluated taxonomically.

**Methods:**

In the present study, several populations of this species complex along with specimens of its closely related taxa in Iran, *T. sanguinolentus, T. ruderatus* and *T. persicus*, were examined using partial nucleotide sequences of two mitochondrial genes (cytb and ND2) (total length 1,322 bp).

**Result:**

Populations of *T. sanguinolentus* clustered within the *T. agilis* species complex, thus indicating its paraphyly, but *T. sanguinolentus* was previously determined to be a species based on morphological features. The *T. agilis* species complex forms two distinct major clades, each of which is represented by several local populations on the Iranian Plateau. At least five distinct taxa can be identified within this traditional group. Our biogeographic evaluation of the molecular dataset suggested that the *Trapelus* complex originated in the Late Oligocene (30 mya) and subsequently diversified during the early to middle Miocene (22–13 mya). At first, the predominantly western clade of *Trapelus ruderatus* diverged from the other clades (22 mya). Afterward, *Trapelus persicus* diverged around 18 mya ago. The broader *T. agilis* complex started to diverge about 16 mya, forming several clades on the Iranian Plateau and in Central Asia. The different lineages within this species complex appear to be the result of vicariance events and dispersal waives. The corresponding vicariance events are the formation of the Zagros and Kopet Dagh basins (16–14 mya), and consequently, the aridification of the Iranian Plateau in the late Miocene (11–6 Mya).

## Introduction

**Figure 1:**
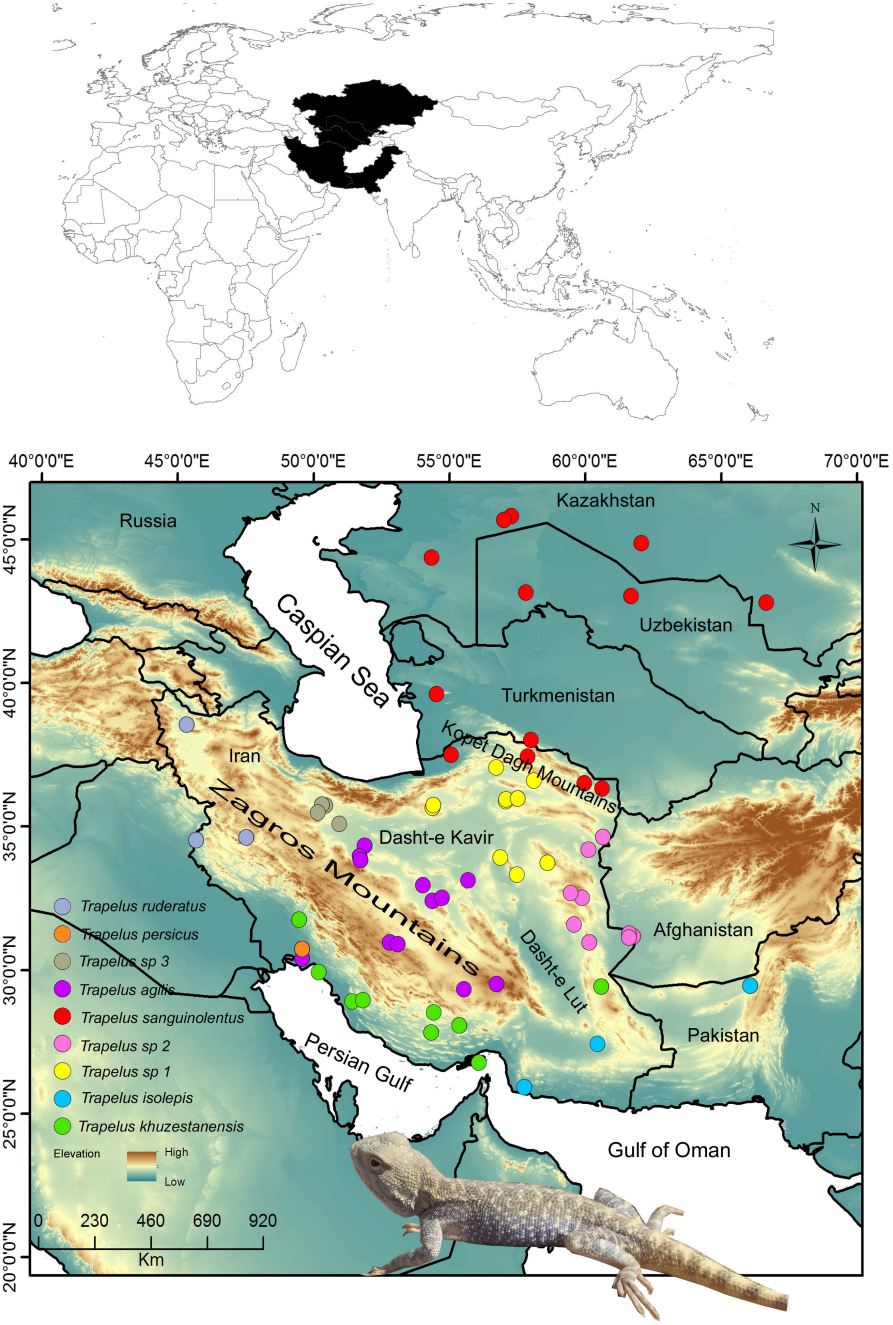
Distribution pattern of examined samples. Map of the study region from Central Asia to the Iranian Plateau in western Asia. Sampling localities for each clade and species are included in the legend and are represented by different colors. Name of clades correspond to the Fig. 2 clades.

The history of the Iranian Plateau extends back to about 40 mya, when the Tethys Sea began to close creating the Alborz Mountains (Alaei, 2006). The formation of the Zagros basin about 15 Mya affected the genetic structure of various terrestrial animals on the Iranian plateau (Smid & Frynta, 2012; Farasat, Akmali & Sharifi, 2016). Many taxa entered Iranian land before the uplifting of the Zagros, Alborz and Kopet Dagh Mountains (Fig. 1) and expanded into the new microhabitats (Smid & Frynta, 2012; Kapli et al., 2015). After the uplifting of these mountain systems, differentiation between populations of a given species was related to adaptation to local ecological conditions (Smid & Frynta, 2012; Ahmadzadeh et al., 2013), causing the appearance of strong geographical barriers and corridors depending on the species habitat type, such as steppe, mountain, or desert.

**Figure 2:**
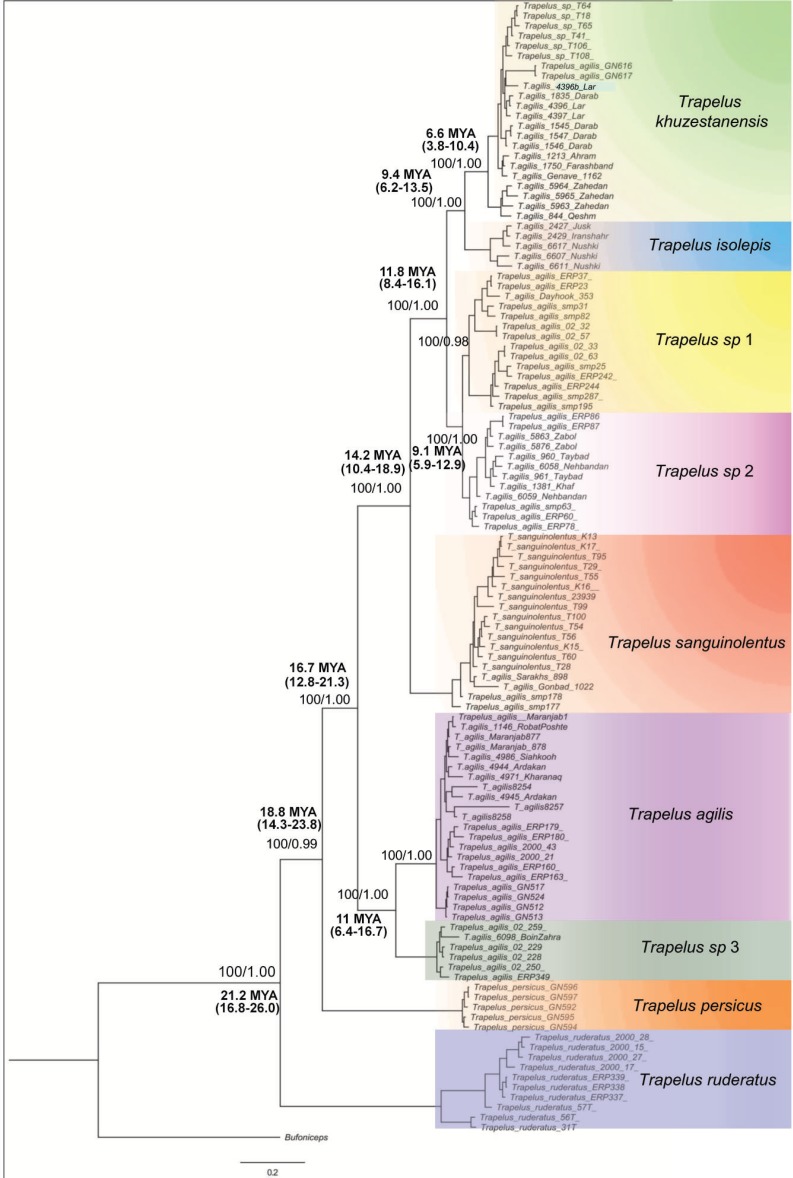
Phylogenetic tree of Trapelus agilis based on mtDNA genes. Bayesian Inference phylogenetic tree of all species of the genus Trapelus in the Iranian Plateau inferred from ND2 and Cyt b mitochondrial gene fragments. Bufoniceps laungwalaensis was used as the outgroup. Numbers next to the nodes present the ML bootstrap and BI posterior probabilities, respectively (ML/BI). Numbers below or above the values indicate the time of divergence in a given node.

The steppe agamas of the genus *Trapelus* are an old clade of agamid lizards with an Afro-Arabian origin (Macey et al., 2000). They have evidently existed in Asia since the Miocene (Macey et al., 2000). Moody (1980) revised the family Agamidae and resurrected the genus *Trapelus* Cuvier, 1816. These lizards are characterized by short, thick heads (Moody, 1980), in contrast to the species of the genus *Agama*, and by deeply sunken tympanums with a few spiny scales around ear opening. The genus *Trapelus* consists of 13 species (Uetz, 2019), which are distributed from northwestern Africa, along the Saharan border, through the Near East to southwest and central Asia (Rastegar-Pouyani, 1999; Rastegar-Pouyani, 2005; Wagner, 2006; Wagner & Crochet, 2009). They constitute one of the major components of the Iranian Plateau and central Asian fauna and are highly adapted to steppe, semi deserts, and desert environments (Macey et al., 2000). Traditionally, four species have been reported on the Iranian Plateau and in central Asia, *T. agilis, T. sanguinolentus, T. persicus and T. ruderatus* (Šmíd et al., 2014). Still, an extensive debate and controversy exists regarding the taxonomic status and phylogenetic relationships among and within these groups (Anderson, 1999; Rastegar-Pouyani, 1999; Rastegar-Pouyani, 2000; Rastegar-Pouyani, 2005; Macey & Ananjeva, 2004; Melville et al., 2009).

*Trapelus agilis* is distributed on the Iranian Plateau and in adjacent regions of southwestern Asia (Šmíd et al., 2014). Within the vast distribution range of *T. agilis*, several distinct subspecies have already been assigned to local populations. Based on extensive morphological work, including work on different populations of *T. agilis* covering almost all of its range, Rastegar-Pouyani (1999) reported four subspecies within this complex group: the nominal subspecies *T. agilis agilis*, occurring on the central and southern Iranian Plateau; *T. a. pakistanensis*, restricted to the lowland and semi desert regions of southeastern Pakistan and to adjoining Indian territory; *T. a. sanguinolentus*, occupying the northern Iranian Plateau, vast areas of central Asia and westward into southeastern Europe, but previously assigned as a distinct species (Bannikov et al., 1977); and *T. a. khuzestanensis* occurring in the southwestern area of the Iranian Plateau and possibly in neighboring regions of Iraq (Rastegar-Pouyani, 1999). *T. a. isolepis* occurs in southeastern Iran and adjoining regions of Pakistan and Afghanistan regions. It has been synonymized with *T. a. agilis* by Rastegar-Pouyani (1999). Based on allozyme data, Macey & Ananjeva (2004) concluded that the Central Asian and European populations should be assigned to a distinct taxonomic entity, *T. sanguinolentus*. The previous morphological systematic studies on this species recognized differentiation among populations of *T. agilis* in Iran (Rastegar-Pouyani, 1999; Rastegar-Pouyani, 2005). Two scenarios were suggested for the variation among populations, such as adaptation to the local conditions and geographic isolation regarding to the orogeny events like the uplifting of the Alborz, Zagros and Kopet Dagh mountains in Iran (Fig. 1) (Rastegar-Pouyani, 1999). In this view, *T. a. khuzestanensis* from southwestern Iran diverged from the other subspecies with the uplifting of the Zagros Mountains in the late Miocene and then other populations diverged from each other to adapt to the local conditions through natural selection (Rastegar-Pouyani, 1999).

In this contribution, we present the comprehensive molecular analysis of the *T. agilis* complex to reveal phylogenetic relationships and intraspecific differentiation within this group, including populations from its range on the Iranian Plateau and some populations from Central Asia. We estimated divergence times of the lineages and their relation to the formation of major mountains and basins in Asia.

## Materials & Methods

### Sampling and DNA extraction

The fieldwork was conducted on the Iranian Plateau and in Central Asia to collect specimens from different populations of *T. agilis* and its closely related species, *T. sanguinolentus*, *T. persicus* and *T. ruderatus*, during 2005 to 2016. Our sampling covers the entire distribution range of *T. agilis* in Iran and of *T. sanguinolentus* from Kazakhstan to Iran (Fig. 1). In total, 114 specimens were included in the study. Tissue samples were obtained from all specimens and voucher specimens were placed in the Sabzevar University Herpetological Collection (SUHC) at Hakim Sabzevari University (Table S1). We received a full approval for this research from National Research Ethics Committee (NREC) (IR.IUMS.REC.1397.085).

Total genomic DNA was extracted from tissue samples (liver, blood or tail tissues) and stored in 96% ethanol following Sambrook, Fritsch & Maniatis (1989). Two mitochondrial (mtDNA) genes, cytochrome b (*Cyt b*) and ND2, were amplified from all samples. The PCR program, primers and length of fragments are summarized in Table S2. We chose different outgroups from closely related species of *Trapelus persicus*, *T. ruderatus* and a sample from the genus *Bufoniceps* as the known sister taxon of the genus *Trapelus* (Pyron, Burbrink & Wiens, 2013).

### Phylogenetic analyses and estimating divergence times

Sequences were aligned using the ClustalW method with default setting as implemented in BioEdit v. 7.0.5 (Hall, 1999). To find the best fit evolutionary model, we used JModelTest v. 2.1.3 (Posada, 2008) according to the Akaike Information Criterion (AIC; Akaike, 1974). The best fit model obtained for each partition within the concatenated dataset was TVM + G for Cyt *b* and TIM + I + G for *ND2*. MEGA 6.0 (Tamura et al., 2013) was used to translate the protein coding sequences into amino acids and to find any stop codons, as well as to compute the un-corrected genetic distance (P-distance) between populations separately in each gene. The program DAMBE 4.1.19 (Xia & Xie, 2001) was used to analyze saturation plots because saturation can influence reliability of results of molecular phylogeny analyses. To reconstruct phylogenetic trees, Maximum Likelihood (ML) and Bayesian Inference (BI) methods were employed. RaxML 7.4.2 (Stamatakis, 2006) implemented in RaxmlGUI 1.3 (Silvestro & Michalak, 2012) were run for ML analysis with default parameters and 1,000 replicates. MrBayes 3.2.1 (Ronquist et al., 2012) was used for BI tree and run for 10^7^ generations with sample frequency of every 1,000 generations. Other parameters, such as number of chains and number of runs, were kept as default. Finally, we discarded the first 25% of all trees as burn-in (Condamine et al., 2015). All of these analyses (ML and BI) were performed on the concatenated dataset from both mtDNA genes.

To evaluate the species boundaries, we used the independent Generalized Mixed Yule Coalescent (GMYC) method (Pons et al., 2006). This method relies on single locus data, and then we used a Bayesian concatenated mitochondrial phylogenetic tree that reconstructed within BEAST v.1.8.2 (Drummond et al., 2012). Outgroups were excluded from the analyses. Information regarding BEAST setting was provided in the next paragraph, when talking on divergence time estimation. The GMYC was performed by R software (R Core Team, 2014) under usage of “splits” package (Species Limits by Threshold Statistics; Ezard, Fujisawa & Barraclough, 2009; package available at http://r-forge.r-project.org/projects/splits).

To estimate the divergence time within Iranian representatives of the genus *Trapelus,* we assembled a dataset containing *Calotes, Phrynocephalus, Xenagama, Acanthocercus, Pseudotrapelus* and *Trapelus*. We used BEAST v. 1.8.2 (Drummond et al., 2012) to estimate divergence time between lineages. First, we used one representative of each genetic lineage to estimate the lineage divergence time. For these analyses, combined mtDNA dataset were examined and evolutionary rates were separated for each gene. For the dating analysis, we used the divergence time of selected nodes from Leaché et al. (2014): split between *Calotes* and *Phrynocephalus* (Normal distribution, mean 62.5, stdev 3.0); split between *Xenagama* and *Psedotrapelus* (Normal distribution, mean 18.0, stdev 1.0); the separation between *Acanthocescus* and *Pseudotrapelus* (mean 15.3, stdev 1.0); and the split between *Xenagama* and *Acanthocercus* (mean 7.3, stdev 2.0) (Tamar et al., 2016). Divergence time within *Trapelus* was estimated using a coalescent method. This analysis shows similar result to the other calibration strategy (Yule tree model). We used a combined dataset divided into two partitions as gene fragments. The evolutionary model was set as TVM + G for Cyt *b* and as TIM + I + G for *ND2*. Clocks were considered as lognormal relaxed. The ucld.mean priors were Uniform (initial 0.0001; lower 0; upper 1.0). Finally, the analyses were run as 3 runs: 5 × 10^7^ generations; 5 × 10^3^ sample frequency and 10% burn-in. All three log files were combined with Tree Annotator to reach a consensus tree.

## Results

In total, 114 individuals of *T. agilis* (80 specimens), *T. sanguinolentus* (18)*, T. ruderatus* (10) and *T. persicus* (5) were investigated. A sequence from *Bufoniceps* was considered as the outgroup. Six sequences (representatives of genera *Calotes, Phrynocephalus, Xenagama, Pseudotrapelus* and *Acanthocercus*) were added into the main database to estimate the divergence time. The dataset consisted of 1322 nt with the concatenated nucleotide sequences of two mitochondrial genes, ND2 (∼970 bp; *V* = 475; *Pi* = 419) and Cyt *b* (∼352 bp; *V* = 246; *Pi* = 181). Graphical saturation tests confirmed the absence of a saturation effect for the combined dataset. The uncorrected p-distances of ND2 and Cyt *b* are presented in Table 1. The highest genetic distance in Cyt *b* accounted for 40.0% between the populations of *Trapelus khuzestanensis* and *Trapelus sp* 2. For ND2, the highest distance of 15.1% was observed between *Trapelus isolepis* and *Trapelus sp* 3. In general, genetic distances among Iranian *T. agilis* populations are relatively high and in the range of distances known for distinct reptile species (see Table 1).

**Table 1:** Genetic distance between clades. Un-corrected genetic distance (p distance) between clades and subclades of Trapelus agilis included in this study inferred from ND2 (below diagonal) and Cyt b (above diagonal).

	1	2	3	4	5	6	7	8	9
1. *Trapelus ruderatus*		15.2	20.7	41.5	21.6	18.0	37.5	18.1	19.0
2. *Trapelus persicus*	22.1		14.9	39.8	18.1	12.4	34.1	14.8	17.2
3. *Trapelus sanguinolentus*	19.4	18.2		38.2	12.3	8.7	31.3	13.7	16.2
4. *Trapelus khuzestanensis*	21.7	19.7	10.5		12.1	34.4	40.0	37.4	38.6
5. *Trapelus isolepis*	20.5	18.8	10.6	6.9		8.2	15.7	16.7	14.1
6. *Trapelus sp* 1	20.7	18.9	9.9	7.7	7.6		26.4	8.4	10.4
7. *Trapelus sp* 2	20.6	18.5	10.0	7.0	6.7	4.2		31.4	32.3
8. *Trapelus sp* 3	20.9	16.3	14.4	15.0	15.1	13.9	14.1		7.5
9. *Trapelus agilis*	20.5	17.5	13.5	12.3	12.4	12.3	12.0	9.5	

The topologies of ML and BI analyses for the concatenated dataset were identical, so only the BI tree is illustrated in Fig. 2. According to the recovered topology, *Trapelus* populations in the area consist of at least six well distinct clades. *Trapelus ruderatus* situated at the base of the tree. *Trapelus persicus,* from southern Iran in Khozestan province, represents the second clade at the base of the tree. Central Asian and northeastern Iranian populations classified as *T. sanguinolentus* that are embedded within the populations traditionally attributed to *T. agilis*. Thus, *T. agilis* becomes paraphyletic with two clades. *Trapelus agilis* species complex is a very heterogeneous clade encompassing several local populations traditionally regarded single species as *T. agilis*. Clades of this species complex are subsequently divided into several clades (*Trapelus khuzestanensis, Trapelus isolepis, Trapelus sp1, Trapelus sp2, Trapelus sp3* and *Trapelus agilis*).

Genetic variability within *Trapelus agilis* is high, as represented in both genetic distance (Table 1) and the GMYC result (Fig. S1). The analyses recovered 10 potative species within the species complex (log *L*_*null*_ = 292.652; log *L*_*GMYC*_ = 294.667; LR 4.02; *p* < 0.133).

Our divergence time estimate (Fig. S2) reveals that the complex originated in the late Oligocene (about 24.2 mya) and subsequently diversified during the early to middle Miocene (22–13 mya). First, the predominantly western clade of *Trapelus ruderatus* diverged from the other clades (22 mya). Then, a splitting event occurred about 18 mya, resulting in the separation of *Trapelus persicus* from the traditional *T. agilis* clade. The ancestor of *T. agilis* and *T. sanguinolentus* started to diverge roughly 16 mya, in turn leading to the formation of several clades on the Iranian Plateau and Central Asia (Fig. 2).

## Discussion

*Trapelus agilis* senso lato is one of the agamid lizards with a wide distribution range from Central Asia, through Afghanistan into the Iranian Plateau, reaching to the western Zagros Mountains (Anderson, 1999). Different morphological studies have been done (Rastegar-Pouyani, 1999; Rastegar-Pouyani, 2005) on this species complex. According to the latest review based on morphological examination, four distinct morphotypes have been identified within the clade: *T. a. sanguinolentus, T. a. khuzestanensis, T. a. agilis* and *T. a. pakestanensis* (Rastegar-Pouyani, 2005). According to our molecular phylogeny, the Iranian populations of this complex can be divided into six genetic lineages (Fig. 2).

*Trapelus sanguinolentus* has been isolated from eastern and southern populations (*T. khuzestanensis, T. isolepis, T. sp.* 1*, T. sp.* 2) for about 12 mya (Fig. 2). The species is isolated from other populations of the genus *Trapelus* by Kopet Dagh Mountains in north eastern Iran. Closing the Paleo-Tethys and creating the Kopet Dagh basin in 14 mya started the divergence of the Central Asian population from Iran ones and then consequently, uplifting the Kopet Dagh Mountains in 4.5 mya differentiated it from other populations. *Trapelus sanguinolentus* has been considered to be a full species (Ananjeva & Tsaruk, 1987; Zhao & Adler, 1993), but, later, Rastegar-Pouyani (1998), Rastegar-Pouyani (1999) and Rastegar-Pouyani (2005) changed its status to the subspecies level as *Trapelus agilis sanguinolentus* because its morphological differences (strongly keeled and spinose scales) cannot warrant classification at the species level*.* Pyron, Burbrink & Wiens (2013) showed that *T. sanguinolentus* is well distinct from *T. agilis*. The authors then concluded that it could be considered as full species*.* Genetic distances of this clade from all other clades of *T. agilis* in Iran are more than 9.9% (for ND2) that clearly support its species status.

Our data provide evidence for a non-monophyly of Iranian *T. agilis*, a fact that has previously not been reported. Two clades (*T. agilis* and *T. sp.* 3) on the central plateau in combination with four clades (*T. khuzestanensis, T. isolepis, T. sp.* 1*, T. sp.* 2) in south, southeast, southwest and east parts of Iran, comprise the large Iranian Plateau group. However, this group is divided into two main clades by *T. sanguinolentus* in the present study. Despite considerable level of similarities in morphology (Rastegar-Pouyani, 2005), their position in the tree and the amount of genetic distance among them suggests another history (Table 1, Fig. 2). These two main groups (South, east and central populations) are isolated from each other by the Kavir and Lut deserts (Fig. 1) on central part of the Iranian Plateau. Gene flow between these clades may have been limited by this severe geographic barrier, but their morphological similarities may be related to the stable climate condition of the central and eastern plateaus after aridification (Zhang et al., 2014). After the uplifting of the Zagros Mountains (22-14 mya), aridification began in the central and eastern plateaus and changed the vegetation type (Zhang et al., 2014). Ancestral populations of *T. agilis* in the area subsequently expanded their range in all parts of the central and eastern portions of the plateau, then the appearance of the Kavir and Lut deserts resulted in isolation of these populations into two main groups of south, southeast and east and central populations under similar climate conditions.

A lineage of *T. agilis* complex created a continuum population from lowland southwest Iran (Khuzestan and Fars provinces) along coastal regions of the Persian Gulf (Hormozgan province) to southeastern Iran and southwestern Pakistan (Fig. 1). This population includes two clades (*T. khuzestanensis, T. isolepis*) that are differentiated from the others in both marker genes; this might be influenced by adaptation to local conditions. The considerable amount of genetic distance between Qom- Saveh and Kerman, Isfahan and Yazd (*T. agilis*) populations are difficult to attribute to just a clinal variation in morphological features. The basal divergence of this group (*T. agilis* and *T. sp.* 3) occurred at about 9.4 mya, reflecting a deep divergence.

## Conclusions

We acknowledge the limitation of our data for making formal changes in taxonomic status of the studied populations, because both genetic markers used in this study are from the single locus mitochondrial DNA. To make a reliable formal changes, further supports from nuclear markers must be sought. However, the findings of the present study strongly suggest that the taxonomic status of populations within the *agilis/sanguinolentus* species complex in the area needs a fundamental revision. Therefore, we suggest that the species complex should be divided into six distinct taxonomic entities as follows:

1. The populations of costal Persian Gulf and southeastern Iran represent a distinct clade. This clade corresponds to one of the subspecies of Rastegar-Pouyani (1999), *T. agilis khuzestanensis* Rastegar-Pouyani (1999) (Type locality: SW Iran, Khuzistan Province, 5 km northwest of Haft-Gel on the road to Shushtar). We suggest that it should be raised to species rank, *T. khuzestanensis* (Fig. 2).
2. Southeast clade includes the population of the type locality of *T. a. isolepis* (Boulenger 1885) (Type locality: between Magas and Bampur, Iran) in both Baluchistan area of Iran and Pakistan. Therefore, this clade would become *T. isolepis* (Fig. 2).
3. Clades from central Khorasan and Semnan, separated from other lineages by relatively high genetic distances, represent a distinct taxonomic entity and deserve specific status. They await proper description based on additional morphological and ecological data. These clades have been marked as *Trapelus sp.* 1 (Fig. 2).
4. Another clade in extreme eastern Iran, in north Baluchistan and in the southern area of Khorasan is another distinct clade representing a taxonomic entity at the species level. Then, we marked it as *Trapelus sp.* 2 (Fig. 2).
5. Population of*T. agilis* is from the Isfahan province on the Central Plateau (Rastegar-Pouyani, 2005; Shahamat, Rastegar-Pouyani & Rastegar-Pouyani, in press), populations from the central plateau make a distinct clade that should be considered as *T. agilis* (Fig. 2).
6. The population from Qom province included within the Qom- Saveh clade inhabits the western area of the range of this species complex in Iran and could be distinguished as a distinct taxonomic entity of *Trapelus*. It awaits proper description based on additional morphological and ecological data. Here, the is clade marked as *Trapelus sp.* 3 (Fig. 2).

In short, our single locus phylogenetic analyses suggest that all of the described subspecies of *T. agilis* in Iran should be raised to the species rank. In addition, two potentially new species of the genus *Trapelus* were discovered in the area. These distinct lineages are geographically isolated. Their morphology is not variable enough (Rastegar-Pouyani, 1999; Rastegar-Pouyani, 2005; Shahamat, Rastegar-Pouyani & Rastegar-Pouyani, in press) to properly reflect their genetic differentiation.

##  Supplemental Information

10.7717/peerj.8295/supp-1 Table S1 Sample locality, specimen codes, and accession numbers

10.7717/peerj.8295/supp-2 Table S2 Primers sequences and PCR programs

10.7717/peerj.8295/supp-3 Figure S1 GMYC result Result of the species delimitation analyses using unique haplotypes of concatenated Cytb and ND2 GMYC tree. A) Ulterametric tree of *Trapelus agilis* with red colored branches indicated lineages delimitated by GMYC. B) Likelihood surface plot that estimated transition between interspecific diversification and intraspecific coalescence generated by GMYC. C) A plot of lineage-through-time provided the intraspecific increase in branching rate by the vertical red line generated by GMYC.

10.7717/peerj.8295/supp-4 Figure S2 BEAST analyses result Species divergence tree that produced by BEAST v. 1.8.2. The scale at the end of tree shows divergence time and divergence time for each lineage represented next to their nodes (by million years ago).

10.7717/peerj.8295/supp-5 Supplemental Information 1 ND2 alignment sequences

10.7717/peerj.8295/supp-6 Supplemental Information 2 Cytochrome b alignment

## Additional Information and Declarations

### Field Study Permissions

The following information was supplied relating to field study approvals (i.e., approving body and any reference numbers): Department of Environment provided permission for field work (number 95/11669).

### Data Availability

The following information was supplied regarding data availability: Data is available at NCBI Genbank and accession numbers are available in Table S1. All sequences are also available in the Supplemental Files.
