# Supplementary material for: Molecular phylogeny and intraspecific differentiation of the Trapelus agilis species complex in Iran (Squamata: Agamidae) inferred from mitochondrial DNA sequences
Source: PeerJ. 2020 Feb 17;8:e8295. doi: 10.7717/peerj.8295 (PMC7032063; doi:10.7717/peerj.8295)
Supplement: Table S1 [file peerj-08-8295-s001.docx]

| **Specimen code** | **taxon name** | **Country** | **Locality** | **ND2** | **Cytb** | **Longitude** | **Latitude** |
| --- | --- | --- | --- | --- | --- | --- | --- |
| no tag | *Pseudotrapelus sinaitus* | Egypt | _ | AB262447 | KP903464 | _ | _ |
| no tag | *Phrynocephalus vlangalii* | China | _ | EF375660 | **_** | _ | _ |
| no tag | *Phrynocephalus vlangalii* | China | _ | EF375651 | _ | _ | _ |
| CAS 205008 | *Calotes versicolor* | Myanmar | _ | DQ289469 | AY572870 | _ | _ |
| CAS 206551 | *Calotes versicolor* | Myanmar | _ | DQ289470 | _ | _ | _ |
| no tag | *Bufoniceps* | _ | _ | _ | _ | _ | _ |
| ERP 339 | *Trapelus ruderatus* | Iran | 44km on the road from Marand to Khouy around Ghapolikh village | MK880781 | MK880694 | 45.32 | 38.53 |
| ERP 338 | *Trapelus ruderatus* | Iran | 44km on the road from Marand to Khouy around Ghapolikh village | MK880782 | MK880695 | 45.32 | 38.53 |
| ERP 337 | *Trapelus ruderatus* | Iran | 44km on the road from Marand to Khouy around Ghapolikh village | MK880783 | MK880696 | 45.32 | 38.53 |
| 57T | *Trapelus ruderatus* | Iran | Kermanshah province | MK880784 | MK880697 | 47.52 | 34.61 |
| 2000_28 | *Trapelus ruderatus* | Iran | 5okm North Kermanshah, Siahdraeh village | MK880785 | MK880698 | 47.52 | 34.61 |
| 2000_27 | *Trapelus ruderatus* | Iran | Ghasr-Shirin , Kermanshah province | MK880786 | MK880699 | 45.68 | 34.52 |
| 2000_17 | *Trapelus ruderatus* | Iran | 5okm North Kermanshah, Siahdraeh village | MK880787 | MK880700 | 45.68 | 34.52 |
| 2000_15 | *Trapelus ruderatus* | Iran | Ghasr-Shirin , Kermanshah province | MK880788 | MK880701 | 47.52 | 34.61 |
| 56T | *Trapelus ruderatus* | Iran | Kermanshah province | MK880789 | MK880702 | 47.52 | 34.61 |
| 31T | *Trapelus ruderatus* | Iran | Kermanshah province | MK880790 | MK880703 | 47.52 | 34.61 |
| GN592 | *Trapelus persicus* | Iran | Around Omidiyeh, Khozestan province | MK880791 | MK880704 | 49.57 | 30.72 |
| GN596 | *Trapelus persicus* | Iran | Around Omidiyeh, Khozestan province | MK880792 | MK880705 | 49.57 | 30.72 |
| GN597 | *Trapelus persicus* | Iran | Around Omidiyeh, Khozestan province | MK880793 | MK880706 | 49.57 | 30.72 |
| GN595 | *Trapelus persicus* | Iran | Around Omidiyeh, Khozestan province | MK880794 | MK880707 | 49.57 | 30.72 |
| GN594 | *Trapelus persicus* | Iran | Around Omidiyeh, Khozestan province | MK880795 | MK880708 | 49.57 | 30.72 |
| T60 | *Trapelus sanguinolentus* | Kazakhstan | _ | MK880796 | MK880709 | 62.07 | 44.85 |
| T56 | *Trapelus sanguinolentus* | Kazakhstan | _ | MK880797 | MK880710 | 54.34 | 44.36 |
| T55 | *Trapelus sanguinolentus* | Kazakhstan | _ | MK880798 | MK880711 | 57.28 | 45.80 |
| K16 | *Trapelus sanguinolentus* | Kazakhstan | _ | MK880799 | MK880712 | 66.67 | 42.79 |
| K15 | *Trapelus sanguinolentus* | Kazakhstan | _ | MK880800 | MK880713 | 57.01 | 45.66 |
| T29 | *Trapelus sanguinolentus* | Turkmenistan | _ | MK880801 | MK880714 | 54.52 | 39.60 |
| T99 | *Trapelus sanguinolentus* | Uzbekistan | _ | MK880802 | MK880715 | 57.82 | 43.13 |
| 23939 | *Trapelus sanguinolentus* | Kazakhstan | _ | MK880803 | MK880716 | 61.69 | 43.01 |
| T100 | *Trapelus sanguinolentus* | Uzbekistan | _ | MK880804 | MK880717 | 58.00 | 38.00 |
| T54 | *Trapelus sanguinolentus* | Kazakhstan | _ | MK880805 | MK880718 | - | - |
| T28 | *Trapelus sanguinolentus* | Turkmenistan | _ | MK880806 | MK880719 | - | - |
| T95 | *Trapelus sanguinolentus* | Kazakhstan | _ | MK880807 | MK880720 | - | - |
| K13 | *Trapelus sanguinolentus* | Kazakhstan | _ | MK880808 | MK880721 | - | - |
| K17 | *Trapelus sanguinolentus* | Kazakhstan | _ | MK880809 | MK880722 | - | - |
| ERP 898 | *Trapelus sanguinolentus* | Iran | Khorasan Razavi province, Sarakhs | MK880817 | _ | 60.61 | 36.31 |
| ERP 1022 | *Trapelus sanguinolentus* | Iran | Golestan province, 47 km from Gonbad to Incheh bron | MK880818 | _ | 55.06 | 37.49 |
| SMP 178 | *Trapelus sanguinolentus* | Iran | North Khorasan province, 10 km west of Shirvan on the road to Bujnourd | MK880847 | MK880753 | 57.87 | 37.42 |
| SMP 177 | *Trapelus sanguinolentus* | Iran | Khorasan Razavi province, 60 km north of Mashhad | MK880845 | MK880751 | 59.96 | 36.50 |
| T108 | *Trapelus khuzestanensis* | Iran | Fars province | MK880810 | MK880723 | - | - |
| T65 | *Trapelus khuzestanensis* | Iran | Fars province | MK880811 | MK880724 | - | - |
| T64 | *Trapelus khuzestanensis* | Iran | Fars province | MK880812 | MK880725 | - | - |
| T106 | *Trapelus khuzestanensis* | Iran | Fars province | MK880813 | MK880726 | - | - |
| T41 | *Trapelus khuzestanensis* | Iran | Fars province | MK880814 | MK880727 | - | - |
| T18 | *Trapelus khuzestanensis* | Iran | Fars province | MK880815 | MK880728 | - | - |
| GN 616 | *Trapelus khuzestanensis* | Iran | 50km east Majed Solaiman, Aroumd Golgir village | MK880823 | MK880729 | 49.47 | 31.75 |
| GN 617 | *Trapelus khuzestanensis* | Iran | 50km east Majed Solaiman, Aroumd Golgir village | MK880824 | MK880730 | 49.47 | 31.75 |
| ERP 1162 | *Trapelus khuzestanensis* | Iran | Bushehr Province, 35 Km SE Deylam | MK880819 | _ | 50.18 | 29.92 |
| ERP 1213 | *Trapelus khuzestanensis* | Iran | Bushehr Province- On the road from Ahram to Khaeez | MK880864 | MK880777 | 51.42 | 28.90 |
| ERP 1545 | *Trapelus khuzestanensis* | Iran | Fars province, Forg village, Dashte Kenar | MK880866 | MK880768 | 55.37 | 28.06 |
| ERP 1546 | *Trapelus khuzestanensis* | Iran | Fars province, Forg village, Dashte Kenar | MK880867 | MK880769 | 55.37 | 28.06 |
| ERP 1547 | *Trapelus khuzestanensis* | Iran | Fars province, Forg village, Dashte Kenar | MK880868 | MK880770 | 55.37 | 28.06 |
| ERP 1750 | *Trapelus khuzestanensis* | Iran | Fars province, 27 Km West of Farashband | MK880869 | MK880774 | 51.82 | 28.95 |
| ERP 1835 | *Trapelus khuzestanensis* | Iran | Fars province, Between Haji Abad and Darab | MK880870 | MK880771 | 54.42 | 28.51 |
| ERP 4396b | *Trapelus khuzestanensis* | Iran | Fars province, Lar | MK880875 | MK880779 | 54.33 | 27.82 |
| ERP 4396 | *Trapelus khuzestanensis* | Iran | Fars province, Lar | MK880873 | MK880775 | 54.33 | 27.82 |
| ERP 4397 | *Trapelus khuzestanensis* | Iran | Fars province, Lar | MK880874 | _ | 54.33 | 27.82 |
| ERP 844 | *Trapelus khuzestanensis* | Iran | Hormozgan province, South of Qeshm Island, Souza | MK880860 | MK880767 | 56.08 | 26.77 |
| ERP 5963 | *Trapelus khuzestanensis* | Iran | Sistan-Baluchistan province, Zahedan- On the road to Cheshm-e Ziarat | MK880882 | MK880772 | 60.58 | 29.42 |
| ERP 5964 | *Trapelus khuzestanensis* | Iran | Sistan-Baluchistan province, Zahedan- On the road to Cheshm-e Ziarat | MK880883 | _ | 60.58 | 29.42 |
| ERP 5965 | *Trapelus khuzestanensis* | Iran | Sistan-Baluchistan province, Zahedan- On the road to Cheshm-e Ziarat | MK880884 | MK880773 | 60.58 | 29.42 |
| ERP 2427 | *Trapelus isolepis* | Iran | Hormozgan province, around Jusk | MK880871 | MK880766 | 57.75 | 25.91 |
| ERP 2429 | *Trapelus isolepis* | Iran | Sistan-Baluchistan province, Iranshahr to Bazman | MK880872 | _ | 60.45 | 27.42 |
| ERP 6607 | *Trapelus isolepis* | Pakistan | Baluchistan province, Nushki | MK880888 | MK880776 | 66.07 | 29.45 |
| ERP 6611 | *Trapelus isolepis* | Pakistan | Baluchistan province, Nushki | MK880889 | _ | 66.07 | 29.45 |
| ERP 6617 | *Trapelus isolepis* | Pakistan | Baluchistan province, Nushki | MK880890 | _ | 66.07 | 29.45 |
| ERP 353 | *Trapelus sp* 1 | Iran | South Khorasan province, Deyhook | MK880816 | _ | 57.48 | 33.32 |
| ERP 37 | *Trapelus sp* 1 | Iran | South Khorasan province, Around Dehgasht village | MK880830 | MK880736 | 58.61 | 33.72 |
| ERP 23 | *Trapelus sp* 1 | Iran | South Khorasan province, Around Dehgasht village | MK880831 | MK880737 | 58.61 | 33.72 |
| SMP 31 | *Trapelus sp* 1 | Iran | Khorasan Razavi province, Around Parvand village 60 km southwest of sabzevar | MK880839 | MK880745 | 57.07 | 35.87 |
| SMP 82 | *Trapelus sp* 1 | Iran | South Khorasan province, 24 km on the road between tabass Bardaskan | MK880849 | MK880755 | 56.87 | 33.90 |
| 02_32 | *Trapelus sp* 1 | Iran | Semnan province, 60Km South Damghan | MK880835 | MK880741 | 54.37 | 35.65 |
| 02_57 | *Trapelus sp* 1 | Iran | Semnan province, 60Km South Damghan | MK880836 | MK880742 | 54.37 | 35.65 |
| 02_33 | *Trapelus sp* 1 | Iran | Semnan province, 60Km South Damghan | MK880837 | MK880743 | 54.41 | 35.73 |
| 02_63 | *Trapelus sp* 1 | Iran | Semnan province, 60Km South Damghan | MK880838 | MK880744 | 54.41 | 35.73 |
| SMP 25 | *Trapelus sp* 1 | Iran | Khorasan Razavi province, Around Parvand village 60 km southwest of sabzevar | MK880840 | MK880746 | 57.09 | 35.94 |
| ERP 244 | *Trapelus sp* 1 | Iran | Khorasan Razavi province, South Sabzevar | MK880841 | MK880747 | 57.51 | 35.97 |
| ERP 242 | *Trapelus sp* 1 | Iran | Khorasan Razavi province, South Sabzevar | MK880842 | MK880748 | 57.51 | 35.97 |
| SMP 195 | *Trapelus sp* 1 | Iran | North Khorasan province, 10 km to Sankhast | MK880843 | MK880749 | 56.72 | 37.05 |
| SMP 287 | *Trapelus sp* 1 | Iran | Khorasan Razavi province, 60km northwest of Sabzevar | MK880844 | MK880750 | 58.10 | 36.59 |
| ERP 86 | *Trapelus sp* 2 | Iran | Sistan-Baluchestan province, Doostmohammad khan village | MK880856 | MK880762 | 61.76 | 31.16 |
| ERP 87 | *Trapelus sp* 2 | Iran | Sistan-Baluchestan province, Doostmohammad khan village | MK880857 | MK880763 | 61.76 | 31.16 |
| ERP 5863 | *Trapelus sp* 2 | Iran | Sistan-Baluchistan province, Zabol, Akbarabad village | MK880880 | _ | 61.61 | 31.25 |
| ERP 5876 | *Trapelus sp* 2 | Iran | Sistan-Baluchistan province, Zabol, Niyatak village | MK880881 | _ | 61.61 | 31.12 |
| ERP 960 | *Trapelus sp* 2 | Iran | Khorasan province, 25 km SW of Taybad | MK880861 | MK880780 | 60.65 | 34.62 |
| ERP 961 | *Trapelus sp* 2 | Iran | Khorasan province, 25 km SW of Taybad | MK880862 | _ | 60.65 | 34.62 |
| ERP 6058 | *Trapelus sp* 2 | Iran | South Khorasan province, Nehbandan - Heydarabad | MK880885 | _ | 60.14 | 30.96 |
| ERP 6059 | *Trapelus sp* 2 | Iran | South Khorasan province, Nehbandan - Heydarabad | MK880886 | _ | 60.14 | 30.96 |
| ERP 1381 | *Trapelus sp* 2 | Iran | Khorasan Razavi province, Khaf- Mozhn abad | MK880865 | MK880778 | 60.12 | 34.18 |
| SMP 63 | *Trapelus sp* 2 | Iran | South Khorasan province, 15 km east Sarbisheh | MK880828 | MK880734 | 59.89 | 32.51 |
| ERP 60 | *Trapelus sp* 2 | Iran | South Khorasan province, 35 Km west Sarbishe | MK880829 | MK880735 | 59.46 | 32.68 |
| ERP 78 | *Trapelus sp* 2 | Iran | South Khorasan province, 55km west Nehbandan | MK880832 | MK880738 | 59.58 | 31.57 |
| ERP 877 | *Trapelus agilis* | Iran | Isfahan province, Kashan, Maranjab, | MK880820 | _ | 51.87 | 34.32 |
| ERP 878 | *Trapelus agilis* | Iran | Isfahan province, Kashan, Maranjab, | MK880821 | _ | 51.87 | 34.32 |
| no tag | *Trapelus agilis* | Iran | Isfahan province, Kashan, Maranjab, | MK880822 | _ | 51.87 | 34.32 |
| ERP 1146 | *Trapelus agilis* | Iran | Yazd Province- 25 Km Northeast of Robat-e-Posht badam | MK880863 | _ | 55.68 | 33.12 |
| ERP 4944 | *Trapelus agilis* | Iran | Yazd province, Near Chak Chak | MK880876 | _ | 54.38 | 32.40 |
| ERP 4945 | *Trapelus agilis* | Iran | Yazd province, Near Chak Chak | MK880877 | _ | 54.38 | 32.40 |
| ERP 4971 | *Trapelus agilis* | Iran | Yazd province, Dareh Anjir | MK880878 | _ | 54.72 | 32.51 |
| ERP 4986 | *Trapelus agilis* | Iran | Yazd province, Siah koh National Park | MK880879 | _ | 54.03 | 32.95 |
| ERP 8254 | *Trapelus agilis* | Iran | Kuzestan province, Hendijan | MN427873 | _ | 49.58 | 30.39 |
| ERP 8257 | *Trapelus agilis* | Iran | Kuzestan province, Hendijan | MN427874 | _ | 49.58 | 30.39 |
| ERP 8258 | *Trapelus agilis* | Iran | Kuzestan province, Hendijan | MN427875 | _ | 49.58 | 30.39 |
| 2000_43 | *Trapelus agilis* | Iran | Fars province, 50km South Abadeh | MK880858 | MK880764 | 52.81 | 30.95 |
| 2000_21 | *Trapelus agilis* | Iran | Fars province, 50km South Abadeh | MK880859 | MK880765 | 52.81 | 30.95 |
| ERP 160 | *Trapelus agilis* | Iran | Kerman province, 4km south Lalehzar village | MK880846 | MK880752 | 56.74 | 29.51 |
| ERP 163 | *Trapelus agilis* | Iran | Kerman province, 20 km on the road from Sirjan to Shiraz | MK880855 | MK880761 | 55.53 | 29.33 |
| ERP 179 | *Trapelus agilis* | Iran | Fars province, 60km on the road from Abadeh to Shiraz | MK880848 | MK880754 | 53.09 | 30.89 |
| ERP 180 | *Trapelus agilis* | Iran | Fars province, 60km on the road from Abadeh to Shiraz | MK880850 | MK880756 | 53.09 | 30.89 |
| GN 517 | *Trapelus agilis* | Iran | Isfahan province, On the road from kashan to Natanz | MK880851 | MK880757 | 51.73 | 33.82 |
| GN 512 | *Trapelus agilis* | Iran | Isfahan province, North Kashan, Abouzaydabad | MK880852 | MK880758 | 51.70 | 33.95 |
| GN 513 | *Trapelus agilis* | Iran | Isfahan province, North Kashan, Abouzaydabad | MK880853 | MK880759 | 51.70 | 33.95 |
| GN 524 | *Trapelus agilis* | Iran | Isfahan province, On the road from kashan to Natanz | MK880854 | MK880760 | 51.73 | 33.82 |
| 02_250 | *Trapelus sp* 3 | Iran | Tehran province, 50km west Tehran, around Estehard town | MK880825 | MK880731 | 50.43 | 35.72 |
| 02_259 | *Trapelus sp* 3 | Iran | Tehran province, 50km west Tehran, around Estehard town | MK880826 | MK880732 | 50.43 | 35.72 |
| ERP 349 | *Trapelus sp* 3 | Iran | 45km Northeast Qom on the road to Tehran around Haus-e- Sultan Lake | MK880827 | MK880733 | 50.95 | 35.08 |
| 02_229 | *Trapelus sp* 3 | Iran | Tehran province, 50km west Tehran, around Estehard town | MK880833 | MK880739 | 50.30 | 35.75 |
| 02_228 | *Trapelus sp* 3 | Iran | Tehran province, 50km west Tehran, around Estehard town | MK880834 | MK880740 | 50.30 | 35.75 |
| ERP 6098 | *Trapelus sp* 3 | Iran | Markazi Province, Bouin Zahra be Saveh | MK880887 | _ | 50.15 | 35.47 |
