# Supplementary material for: Molecular phylogeny and intraspecific differentiation of the Trapelus agilis species complex in Iran (Squamata: Agamidae) inferred from mitochondrial DNA sequences
Source: PeerJ. 2020 Feb 17;8:e8295. doi: 10.7717/peerj.8295 (PMC7032063; doi:10.7717/peerj.8295)
Supplement: Table S2 [file peerj-08-8295-s002.docx]

| **Locus** | **Primer name** | **OR** | **Primer sequence (5’- 3’)** | **Primer source** | **PCR conditions** |
| --- | --- | --- | --- | --- | --- |
| *ND2* | TGlnf | F | GGGACTATTTTGATGGAGTAG | Pinho et al. (2009) | 94º(5’);94º(45”), 56º(45”), 72º(80”) x 35;72(5’) |
|  | TAlar1 | R | GAGGCCCACTAGTTCTGTAGG | Pinho et al. (2009) |  |
|  | Metf6 | F | AAGCTTTCGGGCCCATACC | Pinho et al. (2009) | 94º(5’);94º(45”), 54º(45”), 72º(80”) x 35;72(5’) |
|  | TAlar2 | R | GGTCTTACAGGGGCTAAGG | Pinho et al. (2009) |  |
| *cytb* | Salvi1 | F | TCCAACTACAAAAACCTAATGACCC | Metallinou et al. (2015) | 94º(5’);94º(30”), 48º(45”), 72º(60”) x 35;72º(5’) |
|  | cytb2 | R | CCCTCAGAATGATATTTGTCCTCA | Palumbi et al. (1991) |  |
|  | CMOS-FUF | F | TTTGGTTCKGTCTACAAGGCTAC | Gamble et al. (2008) | 94º (5’);94º(45”), 55º (45”), 72º (70”) x 40;72º (10’) |
|  | CMOS-FUR | R | AGGGAACATCCAAAGTCTCCAAT | Gamble et al. (2008) |  |
|  |  |  |  |  |  |

**References to Table S2.**

Gamble T, Bauer AM, Greenbaum E, Jackman TR. Evidence for Gondwanan vicariance in an ancient clade of gecko lizards. J Biogeogr. 2008; 35: 88–104.

Metallinou M, Červenka J, Crochet P-A, Kratochvíl L, Wilms T, Geniez P, et al. Species on the rocks: Systematics and biogeography of the rock-dwelling Ptyodactylus geckos (Squamata: Phyllodactylidae) in North Africa and Arabia. Mol Phylogenet Evol. 2015; 85: 208–220.

Palumbi, S.R., Martin, A.P., Romano, S., McMillan, W.O., Stice, L., Grabowski, G. The Simple Fool’s Guide to PCR. Department of Zoology Special Publication, University of Hawaii, Honolulu, HI. 1991.

Pinho C, Rocha S, Carvalho BM, Lopes S, Mourao S et al. New primers for the amplification and sequencing of nuclear loci in a taxonomically wide set of reptiles and amphibians. Conserv Genet Resour. 2009; 2: 181-185.
