# Supplementary figures and images for: Molecular phylogeny and intraspecific differentiation of the Trapelus agilis species complex in Iran (Squamata: Agamidae) inferred from mitochondrial DNA sequences
Source: PeerJ. 2020 Feb 17;8:e8295. doi: 10.7717/peerj.8295 (PMC7032063; doi:10.7717/peerj.8295)

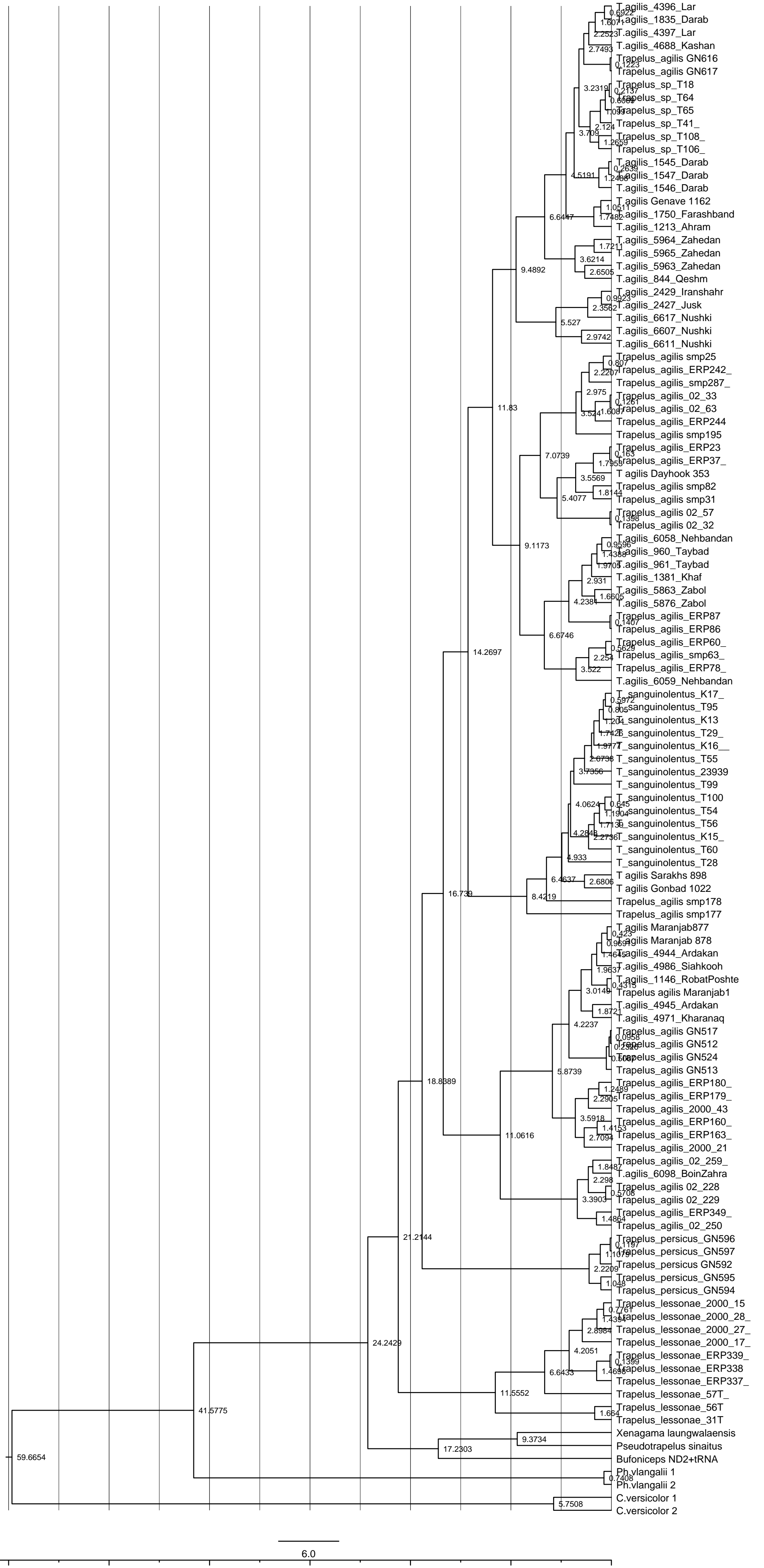

Supplement: Figure S2 — Species divergence tree that produced by BEAST v. 1.8.2. The scale at the end of tree shows divergence time and divergence time for each lineage represented next to their nodes (by million years ago). [file peerj-08-8295-s004.pdf]
